# Supplementary material for: The contribution of temporal coding to odor coding and odor perception in humans
Source: eLife. 2020 Feb 7;9:e49734. doi: 10.7554/eLife.49734 (PMC7007219; doi:10.7554/eLife.49734)
Supplement: Transparent reporting form [file elife-49734-transrepform.docx]

***eLife’s* transparent reporting form**

We encourage authors to provide detailed information *within their submission* to facilitate the interpretation and replication of experiments. Authors can upload supporting documentation to indicate the use of appropriate reporting guidelines for health-related research (see [EQUATOR Network](http://www.equator-network.org/%20)), life science research (see the [BioSharing Information Resource](https://biosharing.org/)), or the [ARRIVE guidelines](http://www.plosbiology.org/article/info:doi/10.1371/journal.pbio.1000412) for reporting work involving animal research. Where applicable, authors should refer to any relevant reporting standards documents in this form.

If you have any questions, please consult our Journal Policies and/or contact us: [editorial@elifesciences.org](mailto:editorial@elifesciences.org).

**Sample-size estimation**

- You should state whether an appropriate sample size was computed when the study was being designed
- You should state the statistical method of sample size computation and any required assumptions
- If no explicit power analysis was used, you should describe how you decided what sample (replicate) size (number) to use

Please outline where this information can be found within the submission (e.g., sections or figure legends), or explain why this information doesn’t apply to your submission:

In the first set of experiments with temporal mixtures we opted for 10 participants per condition, in accordance with sample sizes used in previous studies of similar scope (see Laing et al. 1994, Schriever et al. 2015, Feng et al 2018). In the second set of experiments with temporal mixtures (figure 4 and 6) we increased sample size to 13 per condition. Final sample sizes also depended on the number of participants excluded (see "Data exclusions"). Sample size is clearly stated in each relevant figure’s legend (Figures 2,3,4, and 5 and Supp. Figures 1,2 and 3) as well as in the methods/results section.

**Replicates**

- You should report how often each experiment was performed
- You should include a definition of biological versus technical replication
- The data obtained should be provided and sufficient information should be provided to indicate the number of independent biological and/or technical replicates
- If you encountered any outliers, you should describe how these were handled
- Criteria for exclusion/inclusion of data should be clearly stated
- High-throughput sequence data should be uploaded before submission, with a private link for reviewers provided (these are available from both GEO and ArrayExpress)

Please outline where this information can be found within the submission (e.g., sections or figure legends), or explain why this information doesn’t apply to your submission:

Each experiment was conducted once and each participant took part in a single session. In each 20-trial session, only trials in which all odor pulses were presented fully within inhalations were retained and the rest discarded. A pre-established criterion for participant inclusion was set to a minimum of 14 valid trials out of 20 trials (70% of total trials). In cases where trial number was other than 20, this threshold was adjusted accordingly. 29 (out of 218) participants failed to meet this criterion and were excluded from further analysis. The number of removed trials within the pool of remaining participants amounted to 93 out of 2018 and 253 out of 3072 trials, or 4.6% and 8.2% of events in the CIN/ORG and CTL/DMTS experiments, respectively.

All relevant information is explicitly stated in the methods section.

**Statistical reporting**

- Statistical analysis methods should be described and justified
- Raw data should be presented in figures whenever informative to do so (typically when N per group is less than 10)
- For each experiment, you should identify the statistical tests used, exact values of N, definitions of center, methods of multiple test correction, and dispersion and precision measures (e.g., mean, median, SD, SEM, confidence intervals; and, for the major substantive results, a measure of effect size (e.g., Pearson's r, Cohen's d)
- Report exact p-values wherever possible alongside the summary statistics and 95% confidence intervals. These should be reported for all key questions and not only when the p-value is less than 0.05.

Please outline where this information can be found within the submission (e.g., sections or figure legends), or explain why this information doesn’t apply to your submission:

The statistical methods, their rationale and implementation in the study are detailed in the methods section. All necessary statistical reporting is stated in the results section as well as in the figures and corresponding legends (Figures 2,3,4, and 5 and Supp. Figures 1,2 and 3).

(For large datasets, or papers with a very large number of statistical tests, you may upload a single table file with tests, Ns, etc., with reference to sections in the manuscript.)

**Group allocation**

- Indicate how samples were allocated into experimental groups (in the case of clinical studies, please specify allocation to treatment method); if randomization was used, please also state if restricted randomization was applied
- Indicate if masking was used during group allocation, data collection and/or data analysis

Please outline where this information can be found within the submission (e.g., sections or figure legends), or explain why this information doesn’t apply to your submission:

Participants were randomly assigned to experimental conditions (type of experimental paradigm, odorants used etc.) to meet planned sample sizes. This information is available in the methods section.

**Additional data files (“source data”)**

- We encourage you to upload relevant additional data files, such as numerical data that are represented as a graph in a figure, or as a summary table
- Where provided, these should be in the most useful format, and they can be uploaded as “Source data” files linked to a main figure or table
- Include model definition files including the full list of parameters used
- Include code used for data analysis (e.g., R, MatLab)
- Avoid stating that data files are “available upon request”

Please indicate the figures or tables for which source data files have been provided:

A comprehensive package containing both MatLab code as well as datasets and user instructions has been uploaded. This package is essentially a pipeline allowing to generate part of the figures in the manuscript from raw data into publication-grade graphic figures.
